# When is transformational leadership associated with team commitment? The moderated mediation of intrateam communication and intrinsic motivation among collegiate soccer players

**DOI:** 10.3389/fpsyg.2026.1852751

**Published:** 2026-08-24

**Authors:** Sujin Kim, Inwoo Kim

**Affiliations:** Department of Sports Science Convergence, Dongguk university, Seoul, Republic of Korea

**Keywords:** communication, intrinsic motivation, moderated mediation, team commitment, transformational leadership

## Abstract

**Introduction:**

Given the critical role of interpersonal dynamics in competitive collegiate team sports, understanding the psychological mechanisms that foster athlete loyalty is essential for long-term team success. This study investigated the structural relationship between coaches' transformational leadership and collegiate athletes' affective team commitment, focusing on the mediating role of intrateam communication and the moderating effect of intrinsic motivation.

**Methods:**

Data were collected from 362 male university soccer players in South Korea. Hayes' PROCESS macro (Model 7) was employed to test the proposed moderated mediation model, utilizing 5,000 bootstrap resamples for significance testing.

**Results:**

The results indicated that transformational leadership was positively associated with team commitment both directly and indirectly through intrateam communication. Furthermore, the association between transformational leadership and communication was significantly contingent upon the athlete's level of intrinsic motivation. Simple slope analysis revealed that while the positive association between transformational leadership and communicative dynamics remained positive across all motivation levels, its magnitude was significantly weaker at lower levels (-1 SD) and reached its strongest level at higher levels (+1 SD) of intrinsic motivation. Finally, the integrated moderated mediation model was confirmed, as the strength of the indirect pathway via communication strengthened as motivation rose.

**Discussion:**

These findings suggest that intrinsic motivation functions as a psychological catalyst that is linked to a stronger relationship between transformational leadership and a robust communicative environment, ultimately strengthening the emotional bond between athletes and their teams. The results emphasize the importance of considering individual motivational profiles when evaluating transformational leadership strategies relation to team commitment.

## 1 Introduction

In the competitive landscape of collegiate sports, where the pursuit of elite performance coincides with critical developmental transitions in young adulthood, a team's stability is contingent upon the psychological bond between athletes and the collective. This bond, primarily conceptualized as affective team commitment, represents a profound emotional attachment characterized by an athlete's identification with the team's mission and values, rather than a sense of contractual or social obligation (Schlechter and Strauss, 2008).

According to the foundational work of Mowday et al. (1979), this commitment is more than a passive loyalty defined here as an athlete's ongoing emotional dedication to the team; it is an active relationship involving a strong belief in the organization's goals and a concomitant willingness to exert considerable effort on its behalf. In the context of collegiate athletics, this manifests as a state of mind where dedication stems from the intrinsic meaning and personal identity athletes derive from their membership (O'Neil and Hodge, 2020; Stenling and Tafvelin, 2014).

For student-athletes, who navigate the dual pressures of sports and academics, such commitment serves as a crucial psychological anchor that secures their investment in the team, effectively buffering against exhaustion and reducing the propensity for premature withdrawal (Jung et al., 2026; Pulido et al., 2018).

Cultivating this profound attachment necessitates coaching strategies that transcend transactional management often limited to rigid rules and simple rewards to embrace a leadership paradigm that elevates an athlete's personal and professional identity (Arnold et al., 2001). As emphasized in the seminal work of Bass (1999), transformational leadership functions by fundamentally aligning the interests of the organization with those of its members, thereby motivating followers to achieve performance beyond initial expectations. This leadership framework operates through four distinct yet interrelated dimensions: idealized influence, inspirational motivation, intellectual stimulation, and individualized consideration (Bass and Avolio, 1995). In sports coaching, these behaviors manifest when coaches act as moral role models (idealized influence), articulate a compelling vision (inspirational motivation), empower athletes to question assumptions and view challenges as growth opportunities (intellectual stimulation), and provide tailored mentoring (individualized consideration) (Bass, 1999).

The efficacy of transformational leadership in collegiate sports lies in its ability to transform the athlete's psychological contract with the team. By articulating a compelling vision and providing tailored support, transformational coaches satisfy the fundamental psychological needs of their players, which serves as a prerequisite for deep-seated loyalty (Stenling and Tafvelin, 2014; Vella et al., 2013). Empirical evidence across sporting contexts suggests that such leadership behaviors are robust predictors of affective commitment; when athletes perceive their coach as being invested in their long-term development, they reciprocate with a heightened sense of ownership and a shared collective purpose (Kim et al., 2017). Ultimately, as transformational leaders bridge the gap between individual aspirations and team goals, they anchor the athlete's commitment not through external pressure, but through a genuine identification with the team's identity (Bass, 1999; Kent and Chelladurai, 2001; Mowday et al., 1979).

The structural relationship between transformational leadership, communication, and commitment operates through an integrated social exchange and identity process. Grounded in Social Exchange Theory (SET; Kelley and Thibaut, 1959; Kim et al., 2017), when coaches provide transformational support—such as individualized mentoring and intellectual encouragement—athletes perceive a high-quality exchange, prompting them to reciprocate through increased dedication and pro-social behaviors (Mowday et al., 1979; Ribeiro et al., 2018). Crucially, transformational leadership fosters this reciprocity by shaping athletes' social identity; as Lock et al. (2012) demonstrated in sport contexts, transformational coaching facilitates self-categorization, encouraging athletes to integrate team attributes into their self-concept (i.e., their psychological identity as a team member). This cognitive shift elevates collective identity, leading athletes to prioritize group-level goals over individual interests (Postmes et al., 2001; Shah et al., 2023). Ultimately, affective commitment is not an isolated outcome of leadership, but the product of a unified climate where social exchanges and shared identity reinforce reciprocal dedication.

Ultimately, affective commitment is not an isolated outcome of leadership. Rather, it is the product of a unified climate where social exchanges and shared identity reinforce reciprocal dedication. The transition from a coach's behavior to an athlete's commitment involves a sophisticated internalization of values filtered through the team's social climate (Korek et al., 2010). As Donnelly et al. (2024) suggest, transformational leadership behaviors primarily influence intermediate team dynamics, which subsequently manifest as psychological outcomes. Central to these dynamics is intrateam communication, encompassing both vertical (coach-athlete) and horizontal (athlete-athlete) exchanges (Abu Bakar et al., 2009). As operationalized by Sullivan and Short (2011), this communication is a multidimensional construct comprising acceptance, distinctiveness, and constructive conflict management. While concepts like team cohesion or collective efficacy represent the functional strength of a group, it is through these specific communicative dimensions that functional elements are transformed into a deep, lasting team commitment. Horizontal communication characterized by acceptance fosters a unified environment that encourages genuine emotional investment, while vertical communication concerning strategic goals enables athletes to identify with the group's overarching mission (Kim et al., 2016; Mishra et al., 2016). Specifically, transformational coaching behaviors directly support these communicative processes: individualized consideration cultivates an environment of acceptance and mutual respect, while intellectual stimulation promotes constructive conflict management and open dialogue among teammates (Sullivan and Short, 2011).

While intrateam communication acts as a primary conduit for transformational influence (Donnelly et al., 2024; Kim et al., 2016), the efficacy of this pathway may vary among individuals. The degree to which an athlete engages in the communicative process depends on their unique psychological readiness and motivational state (Grah et al., 2024). This suggests that the meditational effect of communication is likely contingent upon specific boundary conditions rather than being universal. Grounded in Self-Determination Theory, athletes with high intrinsic motivation are inherently more receptive to the autonomy-supportive and informational resources provided through team communication, which in turn amplifies the positive translation of transformational leadership into affective commitment (O'Neil and Hodge, 2020; Deci and Ryan, 2000). A comprehensive understanding of coaching efficacy therefore requires an investigation of how an athlete's internal drive functions as a boundary condition to optimize this structural relationship (Grah et al., 2024).

Athletes with high intrinsic motivation are characterized by a proactive engagement with their environment and a strong desire for competence and autonomy. For these individuals, a coach's transformational behaviors and the resulting open communication channels are perceived as autonomy-supportive resources that facilitate self-growth (Deci and Ryan, 2000; Murillo et al., 2022). This positive perception allows for a more profound internalization of the team's values, thereby strengthening the link between communication and affective commitment (O'Neil and Hodge, 2020; Pulido et al., 2018). As demonstrated by Grah et al. (2024) in recent moderated mediation research, intrinsic motivation functions as a catalytic moderator that amplifies the impact of transformational leadership; intrinsically driven individuals are more likely to engage deeply with the team's communicative structure, viewing it as a pathway for achieving personal and collective excellence (Berestetska, 2016; Jung et al., 2026).

Conversely, athletes lacking such internal drive may perceive the same transformational leadership messages or intensive communication as external pressures or controlling mechanisms rather than autonomous support (O'Neil and Hodge, 2020; Wee et al., 2020). This suggests that the meditational effect of communication is contingent upon the athlete's motivational state rather than being universal (Donnelly et al., 2024; Grah et al., 2024). Without a baseline of intrinsic readiness, the transformational energy provided by the coach may not effectively translate into a deep-seated psychological anchor (Berestetska, 2016; Khan et al., 2022). Therefore, a comprehensive understanding of coaching efficacy in collegiate sports requires a moderated mediation approach to investigate the specific circumstances under which this mechanism is optimized, focusing on how an athlete's intrinsic motivation facilitates the translation of transformational leadership influence into lasting affective commitment (Dong and Zhong, 2022).

**Figure 1:**
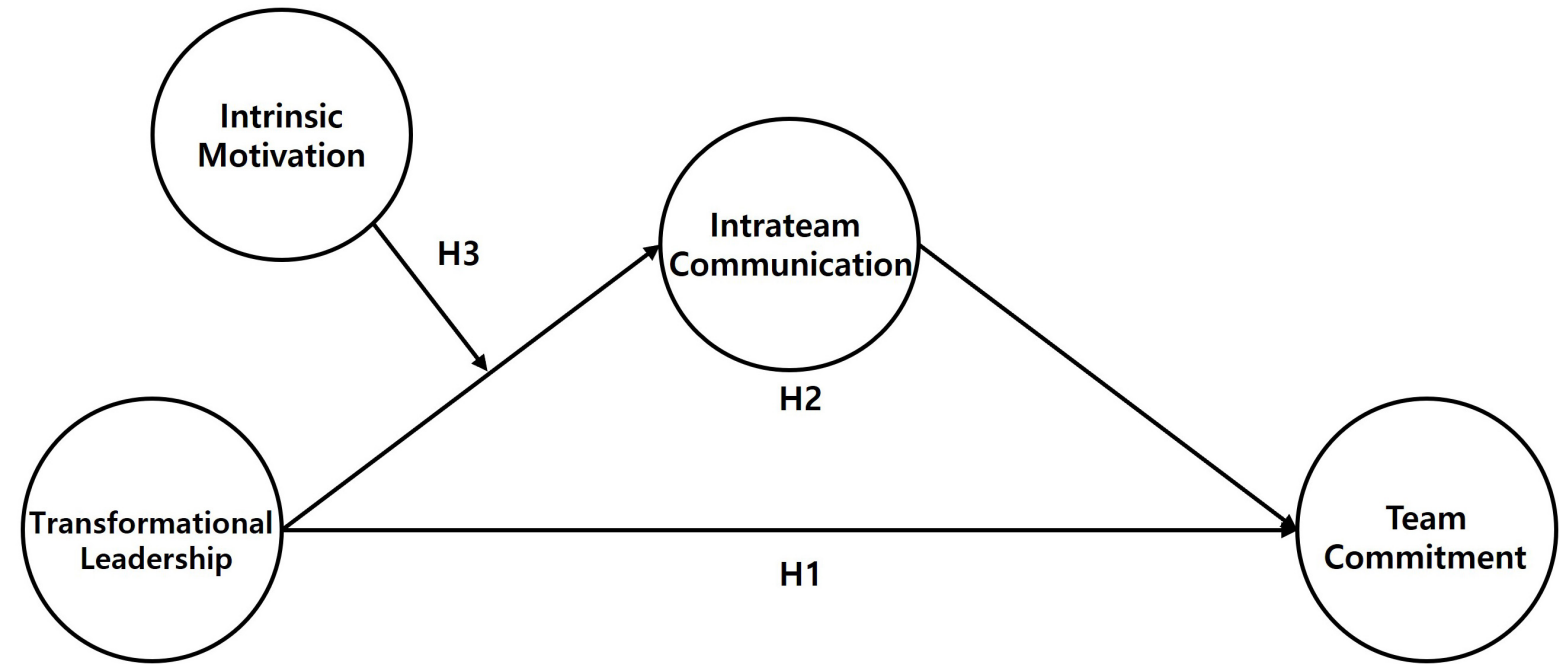
Proposed research model.

Thus, the purpose of this study is to explore the structural relationships between a coach's transformational leadership, intrateam communication, and the affective team commitment of collegiate athletes. Specifically, this research seeks to examine how intrateam communication links transformational leadership behaviors to athletes' psychological attachment, while further investigating whether these associations are contingent upon the athlete's level of intrinsic motivation. By analyzing these variables within a single framework, the study aims to identify the conditional patterns of association that characterize the psychological bond within collegiate sports teams. The hypothesized structural relationships are visually integrated into the research model illustrated in Figure 1.

### 1.1 Research hypotheses

**H1**. A coach's transformational leadership will be positively associated with an athlete's affective team commitment.

**H2**. Intrateam communication will account for a significant portion of the relationship between transformational leadership and affective team commitment, acting as a mediating bridge.

**H3**. The indirect association between transformational leadership and affective team commitment via intrateam communication will be amplified at higher levels of intrinsic motivation.

## 2 Methods

### 2.1 Participants and data source

The data analyzed in this study were derived from a larger research project originally conducted for the first author's doctoral dissertation. The participants were male collegiate soccer players (*M* = 20.420, SD = 1.150, range = 18–23 years) affiliated with the Korea University Sports Federation (KUSF). These athletes compete in the national U-League with the professional goal of transitioning into elite sports careers. This specific demographic was selected to provide a rigorous examination of transformational leadership and team dynamics while minimizing confounding factors such as gender-specific perceptions or cross-sport variations (Kim and Kim, 2026).

A non-probability purposive sampling method was originally employed to recruit active players from various geographical regions and league divisions across South Korea. The target population comprised officially registered student-athletes actively competing in the KUSF U-League. Data collection was conducted from March 3, 2025, to March 17, 2025, through an online survey. From the original dataset, a total of 362 valid responses were retained for the current analysis after excluding incomplete or unreliable data. The sample size (*N* = 362) provides adequate statistical power (exceeding 0.80) to test the hypothesized moderated mediation framework using the PROCESS macro (Faul et al., 2009; Hayes, 2022). In regression-based path analysis, this sample size significantly exceeds the minimum requirements for achieving stable estimates and robust standard errors. Specifically, it provides sufficient statistical power (above 0.80) to detect medium-sized indirect and conditional effects at the 0.05 significance level (Cohen, 1988; Faul et al., 2009). Furthermore, according to Hayes (2022), a sample of this magnitude ensures the reliability of the bias-corrected bootstrapping procedure (with 5,000 or 10,000 resamples), which is the primary method used in this study to test the significance of the moderated mediation index and indirect associations.

The original data collection procedures were reviewed and approved by the Institutional Review Board (IRB) of Dongguk University (Approval No. DUIRB-2025-01-12). Prior to participation, athletes were provided with an informed consent form emphasizing voluntary participation and the right to withdraw at any time without penalty; participants were informed that their involvement offered no monetary compensation but contributed valuable insights toward the advancement of Korean sports. All participants provided informed consent prior to their voluntary participation, and the survey was conducted anonymously to ensure the confidentiality of the student-athletes. For the purpose of the current study, which performs a confirmatory secondary analysis focusing on a moderated mediation framework, the use of this existing dataset was conducted in strict adherence to ethical research standards.

### 2.2 Measurements

To ensure consistency in data collection and minimize participants' cognitive burden, all variables in this study were measured using a 5-point Likert scale, ranging from 1 (strongly disagree/hardly ever) to 5 (strongly agree/almost always).

#### 2.2.1 Transformational leadership

To assess athletes' perceptions of their coaches' transformational leadership style, this study employed the Multifactor Leadership Questionnaire (MLQ-5X) originally developed by Bass and Avolio (1995) and translated and validated in Korean by Jeon (2013). To focus strictly on transformational behavior, non-transformational items (e.g., transactional and passive-avoidant leadership dimensions) were excluded, retaining only the items measuring the four transformational dimensions. The scale was further refined to align with the socio-psychological climate of collegiate sports, substituting organizational terminology with sport-specific terms such as My Coach. For instance, following the intellectual stimulation framework (Bass, 1999), items were modified to reflect practical training and match strategies. The internal consistency of the scale in the current study was Cronbach's α = 0.956.

#### 2.2.2 Intrateam communication

Intrateam communication was evaluated using the Scale of Effective Communication in Team Sports (SECTS-2) developed by Sullivan and Short (2011). This 15-item instrument captures four dimensions of sports-specific interaction: acceptance, distinctiveness, positive conflict, and negative conflict. To ensure linguistic and conceptual equivalence, the original English items were translated and back-translated (Brislin, 1970) by bilingual PhDs in sport psychology. While the original scale utilizes a 7-point format, it was adapted to a 5-point Likert scale for the present study to maintain measurement uniformity. This scale operationalizes communication as a multidimensional social process that reinforces team identity. The reliability coefficient for this scale was Cronbach's α = 0.848.

#### 2.2.3 Team commitment

The athletes' emotional attachment to their teams was measured using the Organizational Commitment Questionnaire (OCQ), originally developed by Mowday et al. (1979). The items were translated into Korean following a forward-back translation procedure (Brislin, 1970) conducted by bilingual sport psychology scholars and adapted to focus on team-level commitment, assessing the degree to which individuals identify with their team's values and their willingness to exert effort for the collective. Consistent with other measures, a 5-point Likert scale was applied. In the current sample, the scale demonstrated high reliability (Cronbach's α = 0.893).

#### 2.2.4 Intrinsic motivation

Intrinsic motivation was assessed using the Korean version of Sport Motivation Scale (SMS-K), translated and validated in the Korean context by Lee (2016). Grounded in Self-Determination Theory, this scale evaluates the inherent satisfaction athletes derive from their sport. This study utilized the intrinsic motivation subscale to examine its role as a psychological boundary condition. The reliability for this subscale was found to be Cronbach's α = 0.940.

### 2.3 Data analysis

The collected data were analyzed using SPSS 26.0 (IBM Corp., Armonk, NY, USA) and the PROCESS macro (v4.2) developed by Hayes (2022). The statistical procedures were conducted as follows: first, data screening procedures were performed; missing data were handled via listwise deletion, and no influential outliers were detected based on Cook's distance values (all <1.0). Regression model assumptions were verified, confirming residual normality (skewness/kurtosis within acceptable limits) and homoscedasticity via the Breusch-Pagan test (*p* > 0.05). Next, descriptive statistics and Pearson's correlation analysis were performed to examine the general characteristics of the variables and the interrelationships between transformational leadership, intrateam communication, affective team commitment, and intrinsic motivation. Cronbach's alpha coefficients were calculated to verify the internal consistency, and discriminant validity was confirmed based on the Fornell–Larcker criterion, where the square root of the Average Variance Extracted (AVE) for each construct exceeded its correlations with other constructs.

Second, to test the hypothesized moderated mediation model, we employed PROCESS macro Model 7. This model allowed for the simultaneous estimation of the mediating process and the moderating role of intrinsic motivation on the first stage of the mediation specifically, the association between transformational leadership and intrateam communication. To mitigate potential multi-collinearity and enhance the interpretability of the interaction terms, all continuous variables were mean centered prior to the analysis.

Third, the statistical significance of the indirect associations and the Index of Moderated Mediation was evaluated using a bias-corrected bootstrapping method with 5,000 resamples. The indirect link was considered statistically significant if the 95% confidence interval (CI) did not include zero. Finally, a simple slope analysis was conducted to further explore the conditional patterns of the relationship between transformational leadership and communication at high (+1 SD) and low (-1 SD) levels of intrinsic motivation (Hayes, 2022).

## 3 Results

### 3.1 Descriptive statistics and participant profile

Prior to the main hypothesis testing, the distributional properties of the collected data were evaluated to ensure their suitability for parametric analysis. Based on the descriptive statistics, the absolute values of skewness and kurtosis for all primary variables (transformational leadership, intrateam communication, affective team commitment, and intrinsic motivation) were found to be within the ranges of −0.909 to.150 and −0.323 to.071, respectively (see Table 1). These results fall well within the conservative thresholds of |3| for skewness and |10| for kurtosis suggested by Kline (2023), confirming that the data set adheres to the assumption of univariate normality.

**Table 1:** Descriptive statistics.

Variables	Mean	SD	Skewness	Kurtosis
Transformational leadership	3.996	0.727	−0.526	−0.141
Intrateam communication	3.646	0.543	0.150	−0.323
Intrinsic motivation	4.311	0.758	−0.909	0.071
Team commitment	3.469	0.609	−0.183	−0.136

Regarding the demographic composition of the 362 male university soccer players, the sample reflects a diverse range of athletic involvement and competitive environments. The participants were active in both U-League 1 (54.4%, *n* = 197) and U-League 2 (45.6%, *n* = 165), representing the comprehensive hierarchical structure of South Korean collegiate soccer. In terms of field roles, the sample was stratified into starters (29.0%), rotation players (34.5%), and substitutes (36.5%), ensuring that the analysis captures the psychological dynamics of players across different levels of participation. The academic distribution was led by freshmen (57.2%), with sophomores (26.0%), juniors (12.2%), and seniors (4.7%) making up the remainder of the cohort. This diverse and balanced profile provides a robust foundation for examining the moderated mediation effects of transformational leadership and communication within a high-pressure sporting context.

### 3.2 Correlation analysis

The Pearson correlation analysis and regression-based diagnostics were conducted to explore the associations between variables and to ensure data suitability for moderated mediation modeling. As presented in Table 2, all primary variables showed significant positive associations with one another (*p* < 0.01).

**Table 2:** Correlation matrix, reliability, and convergent validity.

Variables	1	2	3	4	AVE	CR
1. Transformational leadership	**(0.870)**				0.757	0.925
2. Intrateam communication	0.558^**^	**(0.776)**			0.603	0.784
3. Intrinsic motivation	0.492^**^	0.535^**^	**(0.798)**		0.638	0.943
4. Team commitment	0.644^**^	0.620^**^	0.548^**^	**(0.837)**	0.701	0.873

^**^p < 0.01. AVE, Average Variance Extracted; CR, Composite Reliability. Diagonal elements (in bold with parentheses) are the square root of the AVE; off-diagonal elements are the correlations between variables.

Transformational leadership exhibited a strong positive link with affective team commitment (*r* = 0.644, *p* < 0.01) and was also significantly associated with intrateam communication (*r* = 0.558, *p* < 0.01). Additionally, intrateam communication was significantly correlated with affective team commitment (*r* = 0.620, *p* < 0.01), while intrinsic motivation showed consistent positive associations with commitment (*r* = 0.548, *p* < 0.01), communication (*r* = 0.535, *p* < 0.01), and transformational leadership (*r* = 0.492, *p* < 0.01).

### 3.3 Validity and reliability analysis

Prior to examining the research hypotheses, the structural integrity and psychometric properties of the measurement instruments were rigorously evaluated. To verify the proposed factor structure, a Confirmatory Factor Analysis (CFA) was conducted. The hypothesized four-factor model comprising transformational leadership, intrateam communication, affective team commitment, and intrinsic motivation demonstrated superior fit indices (χ^2^/df = 3.300, CFI = 0.963, TLI = 0.952, RMSEA = 0.080, SRMR = 0.053) compared to more parsimonious nested models.

The analysis supported convergent validity, with Composite Reliability (CR) values ranging from 0.784 to 0.943 and Average Variance Extracted (AVE) values between 0.603 and 0.757, all of which exceeded the recommended thresholds. Furthermore, discriminant validity was confirmed via the Fornell-Larcker criterion; specifically, the square root of the AVE for each construct was consistently greater than its correlation coefficients with all other latent variables (Table 2).

To address the potential for common method bias (CMB) inherent in self-reported data, Harman's single-factor test was performed. The results showed that a single-factor model yielded an extremely poor fit (χ^2^ (76) = 1,626.403, CFI = 0.647, TLI = 0.577, RMSEA = 0.238), indicating that common method variance does not significantly threaten the validity of the current findings (Podsakoff et al., 2003).

Crucially, the statistical diagnostics for multicollinearity confirmed the independence of the variables. All correlation coefficients remained well below the 0.8 threshold, and further analysis revealed that tolerance values were above 0.564 with Variance Inflation Factors (VIF) below 1.773. These results indicate that multicollinearity is not a concern, providing a robust empirical basis for testing the conditional indirect associations in the hypothesized Model 7.

### 3.4 Testing the moderated mediation model

To examine the proposed structural framework, Hayes' (2022) PROCESS macro (Model 7) was employed with 5,000 bootstrap samples. This approach allowed for a simultaneous assessment of the direct associations and the conditional indirect pathways involving intrateam communication and intrinsic motivation. The comprehensive results of the path analysis are summarized in Table 3.

**Table 3:** Results of moderated mediation analysis.

Outcome	Predictor	Coeff	S.E.	*t*	*p*	95% CI
Intrateam communication (*R^2^* = 0.408)	Constant	2.829	0.747	3.789	0.000	(1.361, 4.298)
	Trans leadership (X)	−0.103	0.205	−0.501	0.616	(−0.505, 0.300)
	Intrinsic motivation (W)	−0.081	0.171	−0.475	0.635	(−0.419, 0.256)
	X × W	0.095	0.046	2.082	0.038	(0.005, 0.184)
Team commitment (*R^2^* = 0.514)	Constant	0.331	0.180	1.833	0.068	(−0.024, 0.686)
	Trans leadership (X)	0.429	0.044	9.777	0.000	(0.343, 0.515)
	Communication (M)	0.460	0.054	8.510	0.000	(0.354, 0.566)

The analysis of the direct relationship indicated that a coach's transformational leadership was significantly and positively associated with an athlete's affective team commitment [coeff = 0.429, *t* = 9.777, *p* < 0.001, 95% CI (0.343, 0.515)]. Since the 95% confidence interval did not include zero, this suggests that athletes who perceive their coaches as exhibiting transformational behaviors develop a stronger emotional attachment to their team, even when accounting for the mediating role of communication.

Regarding the mediating mechanism, the analysis revealed a more complex structural relationship than a simple linear path. In the regression model for intrateam communication (Table 3), the unconditional effects of transformational leadership and intrinsic motivation were not statistically significant when the interaction term was included. However, the interaction between transformational leadership and motivation was found to be statistically significant [coeff = 0.095, t = 2.080, p <0.05, 95% CI (0.005, 0.184)], accounting for an additional 0.7% of the variance in team communication (*R*^2^-change = 0.007, *p* < 0.05).

To further clarify the nature of this interaction, a simple slope analysis was performed. The results indicated that the association between transformational leadership and communication was contingent upon the level of internal drive; specifically, the link was more pronounced for athletes with high intrinsic motivation (Effect = 0.371, *p* < 0.001) compared to those with lower levels (Effect = 0.229, *p* < 0.001). In the subsequent path, intrateam communication was significantly and positively linked to higher levels of affective team commitment [coeff = 0.460, *t* = 8.510, *p* < 0.001, 95% CI (0.354, 0.566)]. These findings collectively suggest that while transformational leadership does not function in a vacuum, its influence is effectively channeled through communicative dynamics, particularly when athletes possess the requisite psychological catalyst of intrinsic motivation.

**Figure 2:**
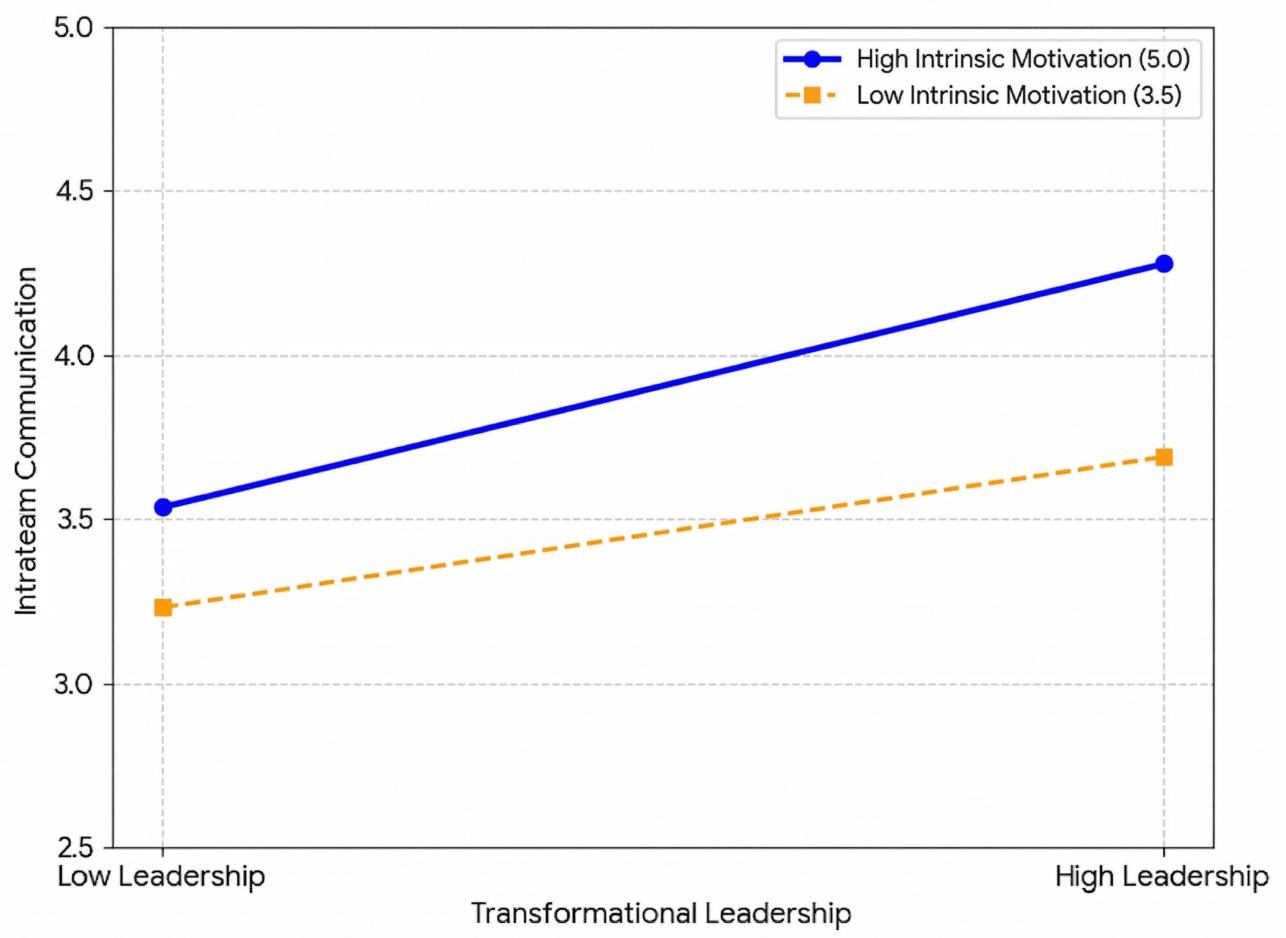
Interaction effect of transformational leadership and intrinsic motivation on intrateam communication. The plot illustrates the simple slopes of transformational leadership on intrateam communication at low (3.5) and high (5.0) levels of intrinsic motivation. The positive association between leadership and communication is significantly amplified when athletes possess higher levels of intrinsic motivation.

As illustrated in Figure 2, the visual representation of the simple slopes further elucidates the nature of this interaction. The slope for athletes with high intrinsic motivation is notably steeper than that for the low-motivation group, indicating that the positive association between a coach's transformational leadership and intrateam communication is amplified when athletes possess a strong internal drive. In contrast, for athletes with lower levels of intrinsic motivation, the communicative benefits derived from transformational leadership appear relatively constrained. These results suggest that intrinsic motivation functions as a psychological catalyst that enhances the leader's ability to foster a robust communicative environment within the team.

Finally, the integrated moderated mediation model was evaluated through the Index of Moderated Mediation and conditional indirect effects. The index was found to be statistically significant [Index = 0.044, BootSE = 0.020, 95% BootCI (0.009, 0.088)]. As shown in the conditional indirect effect analysis (Table 4), the indirect association via intrateam communication was significant at all levels of intrinsic motivation, but its potency increased as motivation rose: low [Effect = 0.105, 95% BootCI (0.054, 0.160)], medium [Effect = 0.149, 95% BootCI (0.099, 0.210)], and high [Effect = 0.171, 95% BootCI (0.113, 0.243)]. These results confirm that intrinsic motivation acts as a psychological catalyst that strengthens the indirect path through which transformational leadership fosters team commitment.

**Table 4:** Conditional indirect effects at levels of intrinsic motivation.

Path	Motivation level	Effect	SE	95% CI
Transformational leadership → Intrateam communication → Team commitment	Low (3.50)	0.105	0.027	(0.054, 0.160)
	Medium (4.50)	0.149	0.028	(0.099, 0.210)
	High (5.00)	0.171	0.034	(0.113, 0.243)
Index of moderated mediation		0.044	0.020	(0.009, 0.088)

## 4 Discussion

The primary objective of this study was to empirically evaluate the structural mechanism linking transformational leadership to affective team commitment through the dual lenses of social exchange and self-determination. Overall, all proposed hypotheses were empirically supported. While prior research has independently established the value of transformational leadership and motivation, the main theoretical novelty of our moderated mediation framework lies in illustrating how these mechanisms conditionally converge within the specific competitive pressures of collegiate soccer.

### 4.1 Empirical validation of transformational leadership as a structural antecedent

The results confirm that transformational leadership serves as a robust predictor of affective team commitment (H1: Supported), providing specific empirical weight to the conceptual frameworks of Bass (1999). Regarding construct measurement, Mowday et al.'s (1979) OCQ was utilized because its focus on value identification and emotional attachment directly aligns with the affective commitment dimension of Meyer and Allen's (1991) three-component model. In the high-pressure environment of South Korean collegiate soccer, our data indicates that transformational coaching does not merely function as a supportive background factor; instead, it acts as a primary structural antecedent that facilitates the alignment of individual aspirations with the collective mission.

Specifically, the strong positive association (coeff = 0.429, *p* < 0.001) suggests that transformational behaviors effectively transcend the constraints of transactional management (Arnold et al., 2001). While transactional paradigms often rely on compliance through rigid rules, our findings imply that transformational coaches facilitate a more profound psychological shift. Bass and Avolio (1995) emphasized, coaches acting as mentors empower athletes to perceive challenges as collective growth opportunities. This study empirically validates this transition, showing that when student-athletes perceive a coach's holistic investment, they move beyond social obligation toward a state of genuine identification (Stenling and Tafvelin, 2014). By satisfying fundamental psychological needs, transformational coaches effectively anchor commitment through internal identification rather than external pressure (Kent and Chelladurai, 2001).

### 4.2 Communication as the operational pathway of social exchange

A pivotal finding of this study is the identified indirect association through intrateam communication (H2: Supported). This result demonstrates that the link between a coach's behavior and an athlete's commitment is not a simple linear sequence, but a sophisticated process of value internalization filtered through the team's social climate (Korek et al., 2010). Our findings reinforce the theoretical model of (O'Neil and Hodge 2020), illustrating that transformational leadership behaviors influence intermediate team dynamics, which are concurrently reflected in profound psychological outcomes.

From the perspective of Social Exchange Theory (SET), our results suggest that the high-quality exchange proposed by Kim et al. (2017) is operationalized through specific communicative dynamics. As coaches foster a climate of acceptance and constructive conflict management (Sullivan and Short, 2011), athletes appear to reciprocate with increased dedication (Ribeiro et al., 2018). In this context, intrateam communication (coeff = 0.460, *p* < 0.001) serves as the social currency that enables reciprocity to manifest. Simultaneously, according to the Social Identity Approach, high-quality communication is what allows athletes to integrate team attributes into their own self-concept (i.e., their subjective view of themselves as team members; Postmes et al., 2001). Our findings highlight that while vertical communication ensures mission alignment, the overall communicative climate is what transforms functional team elements into a lasting emotional bond (Shah et al., 2023).

### 4.3 Intrinsic motivation as a boundary condition for transformational leadership efficacy

The most nuanced insight of this study is the confirmation that the indirect path to commitment is contingent upon the athlete's level of intrinsic motivation (H3: Supported). While communication acts as a link for influence, our significant Index of Moderated Mediation (0.044) provides empirical evidence that this efficacy is not universal but depends on the athlete's psychological readiness (O'Neil and Hodge2020).

Consistent with Self-Determination Theory (SDT), our data reveals that for athletes with high internal drive, transformational behaviors are perceived as autonomy-supportive resources rather than external pressures (Murillo et al., 2022; O'Neil and Hodge, 2020). This allows highly motivated individuals to engage more deeply with the team's communicative structure, viewing it as a vehicle for achieving excellence (Jung et al., 2026). In this sense, intrinsic motivation acts as a “multiplier” that amplifies the transformational leadership-communication link (Effect = 0.371).

Conversely, for athletes lacking this drive, the transformational energy provided by the coach may be perceived as controlling, leading to an attenuated relationship (Effect = 0.228). This underscores that the psychological filter of the athlete (Korek et al., 2010) determines the degree to which transformational leadership influence is successfully internalized. From a cross-sectional perspective, these findings highlight a conditional synergy within the team's climate. For student-athletes navigating dual pressures, intrinsic motivation serves as the essential internal mechanism that facilitates the internalization of values through the communicative process (Pulido et al., 2018). Ultimately, this research clarifies that the most potent psychological attachment is formed when a coach's influence meets an athlete's inherent passion for the sport.

### 4.4 Implications

Intrateam communication serves as facilitator through which transformational leadership influence is associated with commitment. Rather than simply increasing the frequency of interaction, coaches should focus on the qualitative structure of communication, such as fostering an environment of acceptance and constructive conflict management. When athletes feel their voices are heard and disagreements are handled fairly, the coach's vision is perceived as a collective goal rather than unilateral instruction, effectively anchoring the athlete's emotional bond to the team.

Intrinsic motivation acts as a psychological prerequisite, meaning the efficacy of coaching behaviors is contingent upon each athlete's unique psychological state. For athletes with high intrinsic motivation, transformational strategies like intellectual stimulation yield the highest levels of commitment. Conversely, for those with lower internal drive, intensive leadership messages might be misperceived as controlling pressure. In such cases, coaches should first employ autonomy-supportive strategies to rekindle the athlete's inherent interest before expecting deep-seated loyalty.

For collegiate athletes navigating the dual pressures of performance and academics, transformational leadership serves as a crucial psychological anchor. Coaches should adopt a mentor-driven approach that prioritizes an athlete's long-term holistic development over immediate on-field output. When athletes perceive that their growth is genuinely valued, their psychological contract shifts toward a genuine identification with the team's mission. This internal identification is vital for maintaining team stability and reducing the propensity for withdrawal in high-pressure environments.

In conclusion, coaching efficacy in collegiate soccer is optimized when transformational behaviors align with an athlete's inherent passion and a healthy communicative climate. Coaches must recognize that their influence is filtered through these psychological variables and strategically manage both the team's social dynamics and the athletes' internal drive to ensure long-term success.

### 4.5 Limitations and future research

Despite the meaningful insights into the moderated mediation mechanism of coaching efficacy, several limitations should be acknowledged. First, as this study employed a cross-sectional design, the identified relationships represent a structural snapshot of a mutually reinforcing system rather than a definitive causal sequence over time. While the theoretical frameworks of Social Exchange Theory and Self-Determination Theory provide a strong basis for the proposed paths, future research utilizing longitudinal or diary study designs is needed to more accurately capture the temporal dynamics and causal flow between transformational leadership, communication, and commitment.

Second, the sample was limited to male university soccer players in South Korea, which may constrain the generalizability of the findings to other sports, female athletes, or different cultural contexts. The psychological impact of transformational leadership and the nature of intrateam communication can vary significantly depending on the sport's type (e.g., individual vs. team sports) and the gender of the participants. Future studies should replicate this model across diverse athletic populations to verify its broader applicability.

Third, this research relied exclusively on self-reported data from athletes, Specifically, leadership was evaluated based on athletes' subjective perceptions rather than objective leadership behaviors or coaches' self-perceptions. Although athletes' perceptions are critical proximal predictors of psychological outcomes, future investigations should incorporate multi-source or dyadic assessments—such as comparing coach self-reports with athlete evaluations or including objective performance metrics—to mitigate common method bias and enhance empirical rigor.

Finally, while intrinsic motivation was identified as a critical boundary condition, other environmental or individual factors, such as team climate, perceived competence, or the duration of the coach-athlete relationship, might also influence the efficacy of transformational leadership. Future research should explore additional moderators to provide a more comprehensive understanding of the complex psychological landscape in collegiate sports.

## 5 Conclusion

The present study investigated the structural mechanism through which a coach's transformational leadership relates to an athlete's affective team commitment, specifically examining intrateam communication as a mediator and intrinsic motivation as a moderator. The empirical findings offer several meaningful insights into coaching efficacy in collegiate sports.

First, transformational leadership functions as a significant antecedent that helps reinforce the athlete's psychological bond with the collective. The study confirms that coaching behaviors focusing on vision and individual consideration are positively associated with deep-seated loyalty. Second, this association is structurally operationalized through the team's communicative climate. Intrateam communication encompassing acceptance and constructive conflict management functions as a key vehicle that integrates coaching vision into the athlete's value system. Finally, the efficacy of this leadership-communication pathway is not universal but is contingent upon the athlete's motivational state. Intrinsic motivation serves as a catalytic boundary condition, significantly amplifying the indirect effect of transformational leadership on commitment.

In summary, this research illustrates that anchoring affective commitment requires a synergistic alignment between transformational influence and an athlete's inherent psychological readiness. These findings highlight that coaching efficacy is a product of both social dynamics and individual motivational factors, offering a robust empirical foundation for understanding team stability in the high-pressure context of collegiate soccer.

## Data Availability

The raw data supporting the conclusions of this article will be made available by the authors, without undue reservation.
